# Rapid Determination of SARS-CoV-2 Integrity and Infectivity by Using Propidium Monoazide Coupled with Digital Droplet PCR

**DOI:** 10.3390/ijms25116156

**Published:** 2024-06-03

**Authors:** Giuseppe Sberna, Cosmina Mija, Eleonora Lalle, Gabriella Rozera, Giulia Matusali, Fabrizio Carletti, Enrico Girardi, Fabrizio Maggi

**Affiliations:** 1 Laboratory of Virology and Biosafety Laboratories, National Institute for Infectious Diseases “Lazzaro Spallanzani” (IRCCS), 00149 Rome, Italy; 2 Scientific Direction, National Institute for Infectious Diseases “Lazzaro Spallanzani” (IRCCS), 00149 Rome, Italy

**Keywords:** SARS-CoV-2, propidium monoazide, PMA, PMAxx, digital droplet PCR, ddPCR, viral isolation, negative-chain PCR, virus integrity, virus infectivity

## Abstract

SARS-CoV-2 is a highly infectious virus responsible for the COVID-19 pandemic. Therefore, it is important to assess the risk of SARS-CoV-2 infection, especially in persistently positive patients. Rapid discrimination between infectious and non-infectious viruses aids in determining whether prevention, control, and treatment measures are necessary. For this purpose, a method was developed and utilized involving a pre-treatment with 50 µM of propidium monoazide (PMAxx, a DNA intercalant) combined with a digital droplet PCR (ddPCR). The ddPCR method was performed on 40 nasopharyngeal swabs (NPSs) both before and after treatment with PMAxx, revealing a reduction in the viral load at a mean of 0.9 Log copies/mL (SD ± 0.6 Log copies/mL). Furthermore, six samples were stratified based on the Ct values of SARS-CoV-2 RNA (Ct < 20, 20 < Ct < 30, Ct > 30) and analyzed to compare the results obtained via a ddPCR with viral isolation and a negative-chain PCR. Of the five samples found positive via a ddPCR after the PMAxx treatment, two of the samples showed the highest post-treatment SARS-CoV-2 loads. The virus was isolated in vitro from both samples and the negative strand chains were detected. In three NPS samples, SARS CoV-2 was present post-treatment at a low level; it was not isolated in vitro, and, when detected, the strand was negative. Our results indicate that the established method is useful for determining whether the SARS-CoV-2 within positive NPS samples is intact and capable of causing infection.

## 1. Introduction

Severe acute respiratory syndrome-related coronavirus 2 (SARS-CoV-2) is an enveloped positive-sense single-stranded RNA virus, a member of the coronavirus family, and it is the causative agent of coronavirus disease 19 (COVID-19) [1]. The SARS-CoV-2 outbreak was reported in December 2019 from Wuhan, and, since then, over 775 million confirmed cases and over 7 million deaths have been reported globally [1]. Most infected patients show mild or no symptoms [1]; however, many of these test RNA-positive by molecular assays and can remain positive at a low level for a long time, even up to a month, particularly if they are patients with immunocompromised conditions [2,3,4,5]. The main challenges associated with this prolonged SARS-CoV-2 shedding are managing positive patients who must remain isolated until receiving a negative result to prevent the spread of the virus [6], and the correct treatment administration of the immunocompromised patients [7].

To determine whether this prolonged positivity is indicative of the presence of infectious viruses capable of inducing viral replication in the host, the available tests rely on cell cultures, such as viral isolation. Although viral isolation is considered the gold standard of laboratory tests for assessing viral infectivity, it has inherent challenges that make it a complex procedure: it requires experienced personnel, biosafety level 3 laboratories (BSL-3), a long turnaround time, and sensitivity limits that may result in false-negative results in cases of low viral loads [8,9,10]. To address these limitations, molecular tests have emerged that can identify whether a virus is replicative or not [11,12,13,14,15]. These tests detect the negative-chain RNA, which serves as the replicative intermediate of SARS-CoV-2, via a PCR, thus functioning as an indirect marker of active replication [11,12,13,14,15]. Furthermore, a method based on the use of nucleic acid intercalants, i.e., propidium monoazide (PMA) and its derivatives (PMAxx), has been developed to assess the integrity of the virus within clinical samples [16,17,18]. This procedure operates on the principle that PMA can penetrate virions with damaged membranes to bind to viral genomes covalently and irreversibly. This binding prevents amplification by detaching the polymerase when it encounters the intercalant–genome complex. Therefore, only genomes from intact virions will be amplified and detected, while free nucleic acids or those from non-infectious viruses are not considered by a PCR. Initially, this test was primarily used to assess the integrity of bacterial pathogens [19]; however, it has since been applied to numerous viruses [16,17,18,20,21,22,23]. With the emergence of SARS-CoV-2, the question has arisen as to whether the use of PMA can aid in identifying viruses capable of infecting and producing new viral progeny from viral remnants or incomplete viruses and free viral genomes, particularly in samples that test positive by molecular assays but have low viral loads and stay positive for prolonged periods.

This study aims to evaluate the efficacy of PMAxx in vitro using a viral isolate of SARS-CoV-2 via a digital droplet PCR (ddPCR), to apply the established method to clinical samples (i.e., nasopharyngeal swabs (NPSs)), and to assess whether the method correlates with the indicators of active viral replication (negative-chain PCR) and SARS-CoV-2 infectivity/viability (viral isolation).

## 2. Results

### 2.1. In Vitro Settings of PMAxx Treatment

By performing a ddPCR on the serial dilutions of live or UV-inactivated SARS-CoV-2 and treated or not treated (NT) with 50 µM or 200 µM of PMAxx, it was verified that no statistical difference was observed between the treatments with the two concentrations of PMAxx, and that 50 µM of PMAxx was sufficient to bind the virus RNA. In particular, between the used concentrations, there is a minimal mean difference of 0.13 Log copies/mL (standard deviation (SD) ±0.27 Log copies/mL).

**Figure 1:**
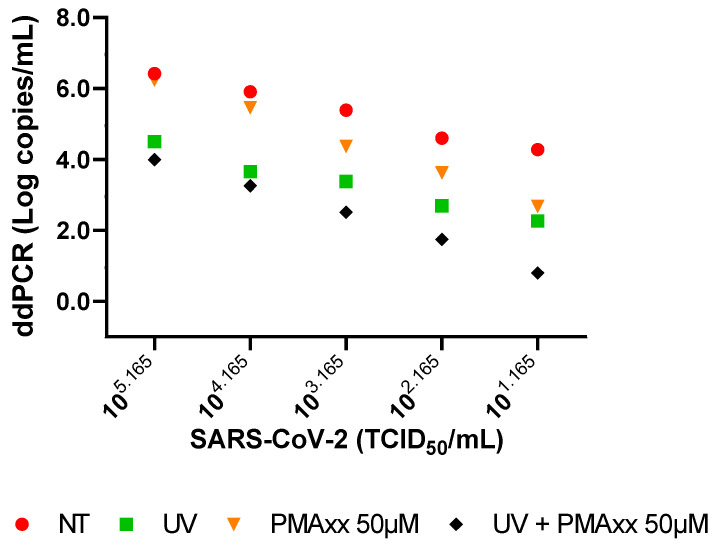
ddPCR results of serial dilutions of SARS-CoV-2 (live and UV-inactivated), both untreated and treated with 50 µM of PMAxx. NT: not treated.

As shown in Figure 1, a reduction in the copy number of viral loads can be observed between the NT and UV-inactivated SARS-CoV-2 samples after the treatment with 50 µM of PMAxx; the detected copy number decreased in both the live and UV-inactivated virus across all the serial dilutions (Figure 1).

### 2.2. Clinical Samples Treated with PMAxx

**Figure 2:**
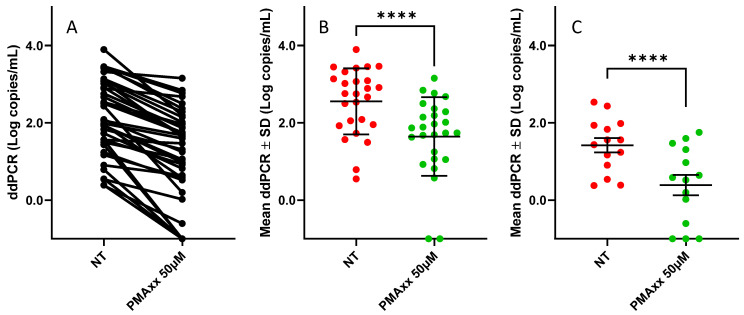
(**A**) ddPCR results of 40 NT and treated with 50 µM of PMAxx NPSs from SARS-CoV-2-positive patients; (**B**) ddPCR results and mean of NT (red spots) and treated with 50 µM of PMAxx (green spots) NPSs from SARS-CoV-2-positive patients with Ct ≤ 25; (**C**) ddPCR results of NT (red spots) and treated with 50 µM of PMAxx (green spots) NPSs from SARS-CoV-2-positive patients with Ct > 25. For graphical representation and statistical analysis, an arbitrary value of −1.0 Log copies/mL was assigned to negative samples. NT: not treated. ****: *p* < 0.0001.

The treatment with 50 µM of PMAxx was conducted on 40 residual NPS samples following the detection of SARS-CoV-2 RNA for clinical purposes. As depicted in Figure 2A, all the samples exhibited a reduction in the copy number after the treatment with PMAxx, with a mean reduction of 0.95 Log (SD ± 0.58 Log copies/mL).

To further investigate the effect of the PMAxx treatment on SARS-CoV-2-positive NPSs, the specimens were categorized based on the cycle threshold (Ct) previously obtained by using the Alinity mSARS-CoV-2 AMP assay. When tested via a ddPCR, the samples with a Ct ≤ 25 exhibited a mean copy number of 2.6 Log copies/mL (SD ± 0.85 Log copies/mL). Following the treatment with 50 µM of PMAxx, the mean copy number decreased to 1.6 Log copies/mL (SD ± 1.0 Log copies/mL), showing a statistically significant difference (*p* < 0.0001; Figure 2B). Regarding the samples with a Ct > 25, the NT-NPSs displayed a mean copy number of 1.4 Log copies/mL (SD ± 0.7 Log copies/mL) via a ddPCR. After the treatment, the mean copy number decreased to 0.4 Log copies/mL (SD ± 0.9 Log copies/mL), also demonstrating a statistically significant difference (*p* < 0.0001; Figure 2C).

### 2.3. Comparison of PMAxx-ddPCR with SARS-CoV-2 Isolation and Negative-Chain PCR

**Figure 3:**
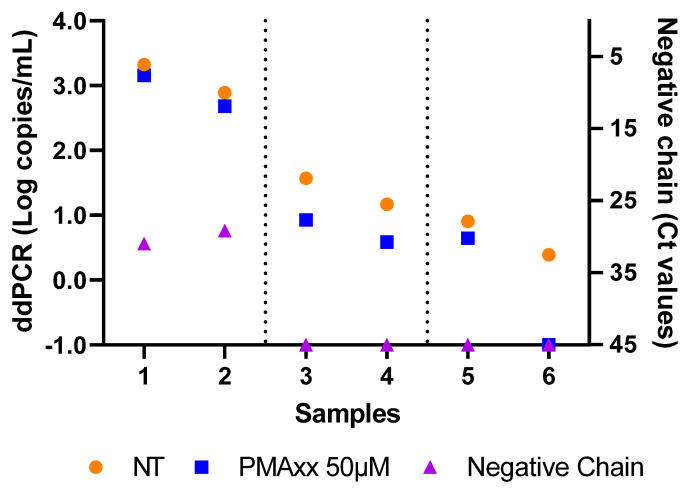
Negative-chain PCR and ddPCR results of six NPSs. For graphical representation, an arbitrary value of −1.0 Log copies/mL was assigned to negative samples. NT: not treated.

Six samples were selected based on the Ct value, as shown in Figure 3; specifically, samples 1 and 2 had Ct values < 20, samples 3 and 4 had Ct values between 20 and 30, and samples 5 and 6 had Ct values > 30. These samples were analyzed to compare the results obtained via a ddPCR (with and without PMAxx treatment) with viral isolation and a negative-chain PCR (Figure 3).

The first two samples exhibited a mean SARS CoV-2 load of 3.1 Log copies/mL without treatment. Upon the treatment with 50 µM of PMAxx, the load underwent a minimal mean reduction of 0.2 Log copies/mL. Both the samples tested positive for the presence of the negative SARS-CoV-2 chain at approximately Ct 30 (Figure 3), and the virus was isolated in in vitro cell cultures when these samples were used as inocula.

Untreated samples 3 and 4 showed a mean viral load of 1.4 Log copies/mL via a ddPCR. When treated with PMAxx, the viral load decreased by a mean of 0.6 Log copies/mL. The SARS-CoV-2 revealed in these samples did not react to the negative-chain PCR, and it was not isolated in the cell cultures (Figure 3). The influence of the background fluorescence amplification could create a quantification bias. The application of new mathematical models designed to improve the sensitivity of the detection in the presence of low viral loads could enable overcoming this gap and obtaining more accurate quantification data [24,25].

Also, SARS-CoV-2 was not isolated in vitro from samples 5 and 6, and both samples reacted negatively when tested for the detection of the negative chain of the virus (Figure 3). Despite these samples both having a viral load below 1.0 Log copies/mL before the treatment and showing similar experimental results, they exhibited different outcomes after the PMAxx treatment: sample 5 revealed a very small difference between the NT and treated (0.3 Log copies/mL) samples, while sample 6 tested negative after 50 µM of PMAxx (Figure 3).

## 3. Discussion

SARS-CoV-2, a highly infectious virus responsible for the COVID-19 pandemic [26], underscores the need for rapid methods to assess the risk of SARS-CoV-2 infection without relying on the cell culture or high-level biosafety facilities. Additionally, the persistent viral positivity in the patient samples, where the virus may lose infectivity during convalescence [9,27], highlights the importance of discriminating between infectious and non-infectious viruses to determine the necessity of isolation and other preventative measures [28,29]. To address these points, a sample pre-treatment with DNA intercalants such as PMA and its derivatives (i.e., PMAxx) has been proposed to distinguish between infectious and non-infectious viruses. Recent studies have demonstrated the selective detection of various viruses using PMA, including norovirus, enterovirus, African swine fever viruses, and Red Sea Bream Iridovirus [20,21,22,23]. Recently, Hong et al. [18] conducted in vitro experiments by inactivating or not inactivating the SARS-CoV-2 with UV light and using sodium dodecyl sulfate as a mild membrane destabilizer to increase the PMA permeability [18]. They effectively demonstrated that a PMA-qPCR can distinguish between the SARS-CoV-2 genome belonging to intact virions and virions with damaged membranes, i.e., infectious and non-infectious, respectively [18]. Another study utilized PMA as a pre-treatment before performing a ddPCR, confirming its effectiveness in distinguishing infectious and non-infectious SARS-CoV-2 [30].

To assess a method that enables the rapid determination of SARS-CoV-2 integrity and infectivity by using a PMAxx-ddPCR, we first performed a ddPCR in vitro on serial dilutions of live or UV-inactivated SARS-CoV-2, treated or NT with 50 µM of PMAxx. We verified the excellent performance of the method and tested the clinical samples to identify the infectious and non-infectious SARS-CoV-2. Additionally, we compared this method with the virus isolation on cell cultures and the presence of the negative SARS-CoV-2 chain by a specific PCR. The established method proved to be effective on the serial dilutions of both the live and UV-inactivated viruses with a pre-treatment of 50 μM of PMAxx.

Applying the method to 40 NPSs, we observed that PMAxx could discriminate between infectious and non-infectious viruses in SARS-CoV-2 patient samples, resulting in a reduction in the viral load at a mean of 0.9 Log copies/mL. Stratifying the samples based on the Ct values (Ct < 25 and >25), we observed significantly different copy numbers before and after the treatment in both groups (*p* < 0.0001).

Furthermore, six samples were stratified based on the Ct values (Ct < 20, 20 < Ct < 30, and Ct > 30) and analyzed to compare the ddPCR results with the viral isolation and a negative-chain PCR. Of these samples, only the two specimens with the highest viral loads after the treatment with PMAxx were isolated and tested positive by a negative-chain PCR, indicating that the PMAxx treatment facilitated the detection of viral copies capable of initiating new replication cycles.

Among the remaining four samples, three specimens were ddPCR-positive after the PMAxx treatment (at a low copy number), tested negative by a negative-chain PCR, and were not isolated. The last sample tested negative via a ddPCR after the PMAxx treatment, and a negative-chain PCR and was not isolated. These findings demonstrate the ability of PMAxx to distinguish between infectious and non-infectious SARS-CoV-2, as evidenced by sample 6, which tested negative via a ddPCR after the PMAxx treatment. In some cases, such as samples 3, 4, and 5, the detected viral genome with a low copy number after the PMAxx treatment may not be infectious but potentially protected by other types of membranes (e.g., double-membrane vesicles [31]) that prevent PMAxx binding. In addition, new mathematical approaches to reduce the quantification bias of certain fluorescence types have been published in several reports that improve the confidence in the interpretation of late-cycle amplification curves, which could clarify the role of the probable background fluorescence amplification at a low viral load [24,25]. These issues can be overcome by performing a negative-chain PCR to verify the active virus replication.

In conclusion, these results indicate that the method involving the pre-treatment with PMAxx and ddPCR is valuable for assessing whether the SARS-CoV-2 within the NPS samples from positive patients is intact and possesses infectious capabilities.

## 4. Materials and Methods

### 4.1. SARS-CoV-2 Stock Preparation and UV Inactivation

The SARS-CoV-2 isolate JN.1 strain hCoV-19/Italy/LAZ-INMI-5946/2023 (GISAID accession ID EPI_ISL_18673911 [32]; EVA-G Ref-SKU: 008V-05662 [33]) was propagated in Vero E6 cells (C1008; African green monkey kidney cells) as previously described [15]. Briefly, cells were maintained in minimal essential medium (MEM) containing 10% fetal bovine serum (FBS) and 0.05 mg/mL gentamycin at 37 °C with 5% CO_2_, and the FBS concentration was reduced to 2% for viral propagation.

The infectious titer of the viral stock used in the study, performed by the Reed and Muench method [34] on VeroE6 cells, was 10^5.165^ TCDI_50_/mL, which was diluted to 10^1.165^ TCDI_50_/mL to perform the analysis with PMAxx (Biotium [35], Fremont, CA, USA).

To verify the PMAxx (Biotium [35], Fremont, CA, USA) activity with the inactivated virus, serial dilutions of SARS-CoV-2 (10^5.165^ TCDI_50_/mL to 10^1.165^ TCDI_50_/mL) were inactivated with UV light for 15 min.

### 4.2. Clinical Specimens

To verify the PMAxx activity in clinical specimens, NPSs were collected from 40 patients hospitalized at the National Institute for Infectious Diseases “Lazzaro Spallanzani” IRCCS in Rome. The median age of patients was 75 years (IQR 63–81), with 24 males (60%) and 16 females (40%). NPSs were put into a sterile tube containing 2–3 mL of viral transport media (Copan UTM^®^ Universal Transport Medium, Copan Diagnostics Italia s.p.a. [36]). Once they reached the laboratory, they were routinely tested for SARS-CoV-2 presence using the Alinity mSARS-CoV-2 AMP assay (Abbott Diagnostics GmbH [37], Wiesbaden, Germany) according to manufacturer instructions [37]. This assay detects RdRp and N genes providing Ct that was used to discriminate samples (less than or greater than Ct 25, as described in Figure 2, and Ct < 20, Ct between 20 and 30, and Ct > 30, as shown in Figure 3). After testing for the presence of SARS-CoV-2, specimens were frozen until the time of PMAxx treatment and ddPCR analysis.

### 4.3. Propidium Monoazide Treatment and Viral RNA Extraction

Propidium monoazide treatment was carried out on 100 µL of clinical specimens and 100 µL of serial dilution of SARS-CoV-2 isolate (10^5.165^ TCDI_50_/mL to 10^1.165^ TCDI_50_/mL), with 50 µM of PMAxx™ Dye (Biotium [35], Fremont, CA, USA). PMA-Lite™ 2.0 LED Photolysis Device (Biotium [35], Fremont, CA, USA) was used to photoactivate PMAxx for 30 min. Importantly, by increasing the concentration used for treatment with PMAxx to 200 µM, the number of copies detected did not change at all, proving that 50 µM is the optimal concentration to perform the treatment.

### 4.4. SARS-CoV-2 Quantification with ddPCR

RNA extraction of both PMA-treated and untreated samples was performed by the QIAamp Viral RNA Mini Kit (Qiagen [36], Hilden, Germany) system, according to the manufacturer’s instructions [38]. Ten µL of RNA per sample were reverse transcribed following the instructions of the TaqMan^®^ Reverse Transcription Reagents (Thermo-Fisher Scientific [39], Norristown, PA, USA) using 25 µM of the N gene reverse primer; the resulting cDNA was purified with the Turbo DNA-free^TM^ Kit (Thermo-Fisher Scientific [39]); after the purification step, the cDNA was quantified using the Bio-Rad QX200 AutoDG Digital Droplet PCR system (Bio-Rad [40], Hercules, CA, USA). Briefly, 900 nM of primers and 250 nM of probe (FAM-labeled) were added to ddPCR Supermix for probes (no dUTPs) (Bio-Rad [40]). To ensure consistent quantification, cDNA from each sample was run in triplicate wells, which were then merged during the analysis. After the PCR reaction, the droplets were subsequently read by a QX100 droplet reader, and the data were analyzed using QuantaSoft software version 1.7.4.0917 (Bio-Rad [38], Hercules, CA, USA). Primers and probe sequences for the N gene were described by Corman et al. [41].

### 4.5. Negative-Chain SARS-CoV-2 RNA PCR

To determine whether the SARS-CoV-2 RNA in samples was associated with active viral replication, we measured levels of antigenomic SARS-CoV-2 RNA (negative chain) because it was considered an indirect marker of ongoing viral replication [11,12,13,14,15]. To measure the negative chain of the N gene, the reverse transcription step was performed in a minus strand-specific manner, based on the use of the N gene forward primer only, as previously described [15]. The resulting cDNA was purified with the Turbo DNA-free^TM^ Kit (Thermo-Fisher Scientific [39]); after the purification step, the cDNA was amplified with SuperScript^®^ III Platinum^®^ One-Step qRT-PCR System (Thermo-Fisher Scientific [39]) with a 25 μL reaction mixture under the following conditions: 0.5 μL of kit enzyme mixture, 12.5 μL of 2 × Reaction Mix, 0.8 μL of MgSO_4_, 0.5 μL of 25 μM primer mix, 0.5 μL of 20 μM of probe, 4.7 μL of nuclease-free water, and 5 μL of cDNA. The following modified thermal profile, omitting the reverse transcription step, was used: 2 min at 95 °C for reverse transcriptase inactivation and DNA polymerase activation, followed by 45 amplification cycles of 15 s at 95 °C and 1 min at 60 °C. Primers and probe sequences for the N gene were described by Corman et al. [41].

### 4.6. SARS-CoV-2 Isolation from Clinical Samples

Viral culture was performed in the BSL-3 facility on Vero E6 cells, as previously described [10]. Briefly, samples were diluted in MEM containing a solution of antibiotics and antimycotics. The mixtures were kept at room temperature for 30 min and inoculated on Vero E6 cells. After 1 h at 37 °C in 5% CO_2_ of incubation, the inoculum was discarded and replaced with MEM containing 2% FBS plus the solution of antibiotics and antimycotics. The cytopathic effect was observed by a light microscope.

### 4.7. Statistical Analysis

Data management and analyses (median, IQR, mean, SD, and *t*-test) were performed using GraphPad Prism version 9.3.1 (GraphPad Software [42], San Diego, CA, USA). A *t*-test was used to evaluate whether the ddPCR results of NT and PMAxx-treated samples were statistically different. For graphical representation and statistical analysis, an arbitrary value of −1.0 Log copies/mL was assigned to negative samples.

## Data Availability

The original contributions presented in the study are included in the article; further inquiries can be directed to the corresponding author.
